# Antibiotic Therapy and Prophylaxis for Snake-Bitten Patients

**DOI:** 10.4269/ajtmh.24-0033

**Published:** 2024-03-19

**Authors:** Hatem Kallel, Jean Marc Pujo, Dabor Resiere

**Affiliations:** ^1^ Intensive Care Unit, Cayenne General Hospital, French Guiana;; ^2^ Tropical Biome and Immunopathology CNRS UMR-9017, Inserm U 1019, Université de Guyane, French Guiana;; ^3^ Emergency Department, Cayenne General Hospital, French Guiana;; ^4^ Intensive Care Unit, Martinique University Hospital, Martinique, France

In a systematic review published in this issue of the *American Journal of Tropical Medicine and Hygiene*, Bonilla-Aldana et al. investigated the prevalence and characteristics of snakebite-related infections.^1^ Indeed, snakebite is a frequent, disabling, and sometimes deadly disease. Most snakebites occur in low, and middle-income countries and affect poor communities with limited access to medical care. Antivenom is the cornerstone of snakebite management.^2^ However, patients may suffer from infectious complications, influencing their outcomes.^3^ Envenoming-related tissue injuries favor microbial proliferation, increasing the likelihood of infection and tissue damage.^4^ Considering that microorganisms from the snake’s mouth are inoculated at the bite, some authors question the effectiveness of prophylactic antimicrobial use in preventing secondary infection.

According to the new report from Bonilla-Aldana et al., secondary infection after snakebite occurred in 32% in Asia, 21% in the Americas, and 29% in Africa.^1^ Therefore, antibiotics are useless in almost 70% of cases. Moreover, many authors have shown that administering prophylactic antibiotics in snake-bitten patients is ineffective in preventing infection,^5^^–^^8^ but may promote bacterial resistance.

To improve management, empiric antibiotics should be directed toward patients at particular risk or suspected of snakebite-related infection. Houcke et al. demonstrated that tissue necrosis, rhabdomyolysis, and thrombocytopenia can predict secondary infection after snakebite.^9^ Local envenoming signs should not be confused with infectious manifestations. However, snakebite-related infection can be suspected in the case of frank local inflammatory symptoms, and this presentation justifies empiric antibiotic prescription. A definite diagnosis of infection can only be made and targeted treatment provided when the responsible microorganism is isolated.

International consortiums recommend amoxicillin–clavulanate (AMC) to prevent infection after an animal bite.^10^^,^^11^ These recommendations are based on studies involving dog, cat, and human bites, but not snakes.^10^^,^^11^ Indeed, snake oral microbiota differ from those of other animals, making AMC ineffective against the involved bacteria most of the time.^12^ Studies on the oral microbiota of snakes^12^ and causal bacteria of snakebite-related infection, including the new report from Bonilla-Aldana et al.^1^ show that isolated microorganisms are mostly *Enterobacteriaceae*, mainly *Morganella morganii*, nonfermentative bacteria such as *Pseudomonas* spp. and *Aeromonas hydrophila*, or Gram-positive cocci such as *Staphylococcus aureus* and *Enterococcus faecalis*. These bacteria usually originate from the snakes’ mouth, contaminated by the fecal flora of prey, which may defecate while being ingested, or from the environment (e.g., *A. hydrophila*, especially in wet areas).^12^ In most cases, the pathogenic bacteria are naturally resistant to AMC.^9^^,^^12^ In addition, in a well-designed study by Sachet et al.^5^ in the Amazon region, AMC showed poor efficacy in preventing secondary infection from snakebites. Accordingly, first-line AMC is not an appropriate option to treat snake-bitten patients, except when guided by microbiological results. Unfortunately, prophylactic AMC is still largely prescribed in this context.^9^^,^^13^

From a microbiological point of view, most *M. morganii* strains are naturally susceptible to piperacillin, ticarcillin, third- and fourth-generation cephalosporins, carbapenems, aztreonam, fluoroquinolones, aminoglycosides, and chloramphenicol.^14^ These antibiotics are also effective against most *Enterobacteriaceae*, including *Escherichia coli*, *Klebsiella pneumoniae*, and *Proteus mirabilis*. *Aeromonas hydrophila* are naturally susceptible to third-generation cephalosporins, piperacillin-tazobactam, ciprofloxacin, and amikacin.^15^^,^^16^ *Enterococcus faecalis* is typically susceptible to non-cephalosporin β-lactams and vancomycin, which represent therapeutic mainstays.^17^ *Staphylococcus aureus* is naturally susceptible to many antibiotics, including oxacillin, first-generation cephalosporins, linezolid, trimethoprim-sulfamethoxazole, gentamicin, clindamycin, ofloxacin, tetracycline, and erythromycin.^18^ Anaerobes are rarely isolated in secondary infection after snakebite. Although piperacillin/tazobactam, ciprofloxacin, clindamycin, or third-generation cephalosporin are active against a wide range of anaerobes, metronidazole is the drug of choice in case of identification of such bacteria or clinical signs suggestive of their involvement.^19^^,^^20^ Anaerobic soft-tissue infections usually develop hours or days after the bite, mainly in devitalized tissues. They can present as collections and crepitus at the bite site.

Considering the frequently isolated bacteria in snakebite-related infection, piperacillin/tazobactam, ciprofloxacin, or a third-generation cephalosporin are the most appropriate antibiotics for empiric therapy. After identification of the causal bacteria, antibiotics must be changed to narrower spectrum drugs based on the microbiological results. Little information is available to guide the optimal duration of antibiotic therapy or the value of combination drug therapy. Available data report antibiotic use for 7 to 10 days with several proposed therapeutic regimens without documented advantages of one strategy over another.^9^^,^^21^

In conclusion, the study by Bonilla-Aldana et al.^1^ accurately shows the prevalence and microbiological patterns of snakebite-related infections. On the basis of the international literature, there is no evidence for the effectiveness of routinely administered prophylactic treatment. Nevertheless, empiric antibiotics can be used in targeted cases with high suspicion of infection, mainly those with necrosis or local signs suggestive of cellulitis. The most appropriate empiric antibiotics are piperacillin/tazobactam, ciprofloxacin, or a third-generation cephalosporin. First-line AMC should no longer be considered because it is ineffective against most bacteria involved in snakebite-related infection. Once the microbiological results are available, deescalation is necessary to provide the narrowest spectrum drug active against the responsible microorganism. Finally, efficiently managing snakebite envenoming requires not only safe and effective antivenom but also appropriate antibiotics, both of which are, unfortunately, often unavailable in poor and disadvantaged regions.
